# Multi-Sensor Data Fusion for Real-Time Surface Quality Control in Automated Machining Systems

**DOI:** 10.3390/s18124381

**Published:** 2018-12-11

**Authors:** E. García Plaza, P. J. Núñez López, E. M. Beamud González

**Affiliations:** 1 Department of Applied Mechanics & Project Engineering, Institute for Energy Research and Industrial Applications (INEI), Higher Technical School of Industrial Engineering, University of Castilla-La Mancha, Avda. Camilo José Cela, s/n, 13071 Ciudad Real, Spain; eustaquio.garcia@uclm.es; 2 Department of Applied Mechanics & Project Engineering, Mining and Industrial Engineering School, University of Castilla-La Mancha, Plaza Manuel Meca 1, 13400 Almadén (Ciudad Real), Spain; elenamaria.beamud@uclm.es

**Keywords:** surface quality control, multi-sensor data fusion, cutting forces, vibration, acoustic emission, signal feature extraction methods, predictive modeling techniques

## Abstract

Multi-sensor data fusion systems entail the optimization of a wide range of parameters related to the selection of sensors, signal feature extraction methods, and predictive modeling techniques. The monitoring of automated machining systems enables the intelligent supervision of the production process by detecting malfunctions, and providing real-time information for continuous process optimization, and production line decision-making. Monitoring technologies are essential for the reduction of production times and costs, and an improvement in product quality, discarding the need for post-process quality controls. In this paper, a multi-sensor data fusion system for the real-time surface quality control based on cutting force, vibration, and acoustic emission signals was assessed. A total of four signal processing methods were analyzed: time direct analysis (TDA), power spectral density (PSD), singular spectrum analysis (SSA), and wavelet packet transform (WPT). Owing to the nonlinear and stochastic nature of the process, two predictive modeling techniques, multiple regression and artificial neural networks, were evaluated to correlate signal parametric characterization with surface quality. The results showed a high correlation of surface finish with cutting force and vibration signals. The signal processing methods based on signal decomposition in a combined time and frequency domain (SSA and WPT) exhibited better signal feature extraction, detecting excitation frequency ranges correlated to surface finish. The artificial neural network model obtained the highest predictive power, with better behavior for the whole data range. The proposed on-line multi-sensor data fusion provided significant improvements for in-process quality control, with excellent predictive power, reliability, and response times.

## 1. Introduction

Current quality control techniques require slow and costly measurement procedures for inspecting finished products. In industrially competitive sectors, these aspects impose serious constraints directly affecting the benefits obtained. Machining monitoring systems are an ideal tool for overcoming these deficiencies, since they permit the real-time monitoring and control of the cutting process, detect in-process malfunctions, and apply corrective measures to avoid the manufacture of defective products. Several aspects of machining processes can be supervized using monitoring techniques. In recent years, numerous publications have focused on the analysis of tool condition [1,2,3,4] and chatter [5,6,7,8], whereas other aspects such as surface finish [9,10,11,12], dimensional precision [11,12,13,14], and chip formation [15,16] have received less attention. The appropriate selection of sensors is crucial for monitoring techniques to be efficacious.

The dynamic character of machining processes, characterized by random and transitory phenomena, has prompted the need for processing on-line information captured by cutting force sensors [15,17,18], mechanical vibration [19,20,21], acoustic emission [22,23,24,25], sound [26,27,28], power consumed [29,30], among others. Frequently, the information from one single sensor has been insufficient for the accurate characterization of a process, underscoring the need for multi-sensor data fusion. The first attempts at using multi-sensor data fusion for process monitoring were developed in the 1990s to monitor tool wear in turning [31,32] and drilling operations [33,34]. Since then, numerous works with sensor fusion have been published [35,36,37,38,39]. The amount of information provided by a sensor depends mainly on the signal feature extraction method. Signal processing techniques in one single domain have been extensively employed, with the time direct analysis (TDA) method [40,41,42,43] being used for analysis in the time domain; and the fast Fourier transform (FFT) method [19,40,43,44,45], and the power spectral density (PSD) method [45,46,47,48] for frequency analysis. In many cases, these methods are not sufficiently efficacious for extracting signal information, which stresses the need for applying more complex analysis techniques that decompose a signal into an independent time series with defined frequency ranges such as: singular spectrum analysis (SSA) [19,49,50], and wavelet packet transform (WPT) [6,15,51]. Statistical parametric characterization is the most common technique used for signal information extraction [21,52]. Moreover, the selection of an efficacious predictive technique is vital for obtaining a high level of precision in predicting data from monitoring systems, with multiple regression (MR) techniques [52,53], artificial neural networks (ANN) [19,54], and support vector machines (SVM) [12,55] being the most frequent methods.

The mean deviation of the assessed profile (*Ra*) is the primary parameter used for the monitoring of surface quality in machining processes. This parameter is an indicator of the surface quality of a product and the behavior of the cutting process, since it is directly linked to machining aspects such as: cutting parameters, tool geometry, use of cutting fluids, tool wear, and chatter, among others [56,57]. In recent years, several surface finish monitoring techniques have been developed. Cutting force and vibration sensors are the most widely employed, and off-line parameters have been incorporated as an additional information source of the cutting process. When one works under ideal conditions—i.e., those recreated in the laboratory—these off-line parameters raise the performance of predictive models. However, under real working conditions characterized by the appearance of random and transitory phenomena, monitoring systems with off-line parameters are more rigid and may mask malfunctions or severe process deficiencies that go undetected by the system.

TDA is the signal analysis method most extensively used by researchers for the monitoring of surface finish. Hessainia [41] used TDA processed vibration signals and cutting conditions for the monitoring of the parameter *Ra*, using a small sample of 27 data built for regression models and validation with the same data. Kirby and Chen [58] used a single component of vibration signals and cutting conditions to monitor the parameter *Ra*. The vibration signal was processed by TDA, using 87 data for fuzzy logic predictive models and validating only with seven workpieces selected under non-random cutting conditions. Upadhay et al. [59] also monitored surface finish using TDA processed vibration signals. In this study 15 workpieces were simultaneously used for the building and validation of the predictive models. Risbood et al. [13] evaluated the parameter *Ra* using TDA processed radial vibration signals and cutting conditions on 20 workpieces to validate ANN models. Özel et al. [60] monitored surface finish using a combination of cutting forces, cutting parameters, cutting time, consumed electrical power, and specific force. The cutting force signals were processed by the TDA method, with only 18 workpieces for building ANN models and 9 for validation.

Signal analysis in the frequency domain is seldom used for the monitoring of surface finish. Abouleta and Mádl [47] calculated the *Ra* parameter with vibration signals, cutting conditions, and tool and workpiece geometry features. The signals were processed by the PSD method using a total of 480 workpieces to build MR models without validating them. Wang et al. [45] calculated the vibration modes of cutting tools by applying the PSD method to cutting forces, and found high frequency vibration (14 kHz) had a significant impact on surface roughness (*Ra*). Moreover, Krolczyk et al. [48] applied the PSD method to 3D measurement surfaces images to analyze the performance of surface morphology in turning operations. Botcha et al. [61] applied frequency analysis to determine the frequency ranges with significant information correlated to surface finish in cylindrical plunge grinding processes.

In contrast, advanced processing methods working simultaneously in a combined time and frequency domain (hereafter, time–frequency domain) are not commonly used owing to their greater complexity. Salgado et al. [49] used SSA processed vibration signals, cutting conditions, and tool angle and radius to estimate the parameter *Ra*, with 35 data for the SVM predictive models, and 20 for validation. García and Núñez [50] applied SSA to vibration signals to monitor surface finish using 270 data test to build MR predictive models, and 90 for validation. Likewise, García and Núñez [52,53] applied the WPT method to vibration and cutting force signals, respectively.

In the last three decades, acoustic emission and sound signals have been commonly applied to the monitoring of tool condition [31,32,62,63]; however, only a few studies have analyzed the monitoring of surface finish. Azouzi and Guillot [14] estimated the parameter *Ra* with the fusion of cutting force, vibration, acoustic emission signals, and cutting parameters. The signals were processed with the TDA method with only 16 workpieces for the ANN models and 5 for validation. Acoustic emission were found to have no significant impact on surface finish. Carou et al. [27] estimated surface finish using TDA processed sound signals and cutting parameters by applying regression models with 18 experimental and 18 validation replicated trials. Frigieri et al. [64] undertook a similar study, except the sound signal was processed in the time-frequency domain using the mel-frequency cepstral coeffiecients, with 15 trials replicated 10 times using 80 for Gaussian mixture models, and validation on non-independent replicated trials. Certain studies have sought to determine the behavior patterns of the acoustic emission signal in relation to surface finish without predictive models. The study of Bhuiyan et al. [42] found the *AE* signal had a moderate correlation with the *Ra* parameter, whilst Pawade and Joshi [65] observed a moderate correlation between the *AE* signal and surface finish with high quality obtained in this study. Rao et al. [66] processed vibration, cutting forces, and acoustic emission signals for the monitoring of surface roughness in ultraprecision diamond turning with a nonparametric clustering technique called, the mean-shift algorithm, and found only the force and vibration signals in the feed direction were adequate for detecting changes in process dynamics and were sensitive to surface variations.

The present study assessed a novel multi-sensor data fusion system for surface quality control in automated machining processes using cutting force, vibration, and acoustic emission sensors. An exhaustive analysis of signal feature extraction methods was performed using two signal processing methods in one single time (TDA) or frequency (PSD) domain; and two processing methods (SSA and WPT) working in the time–frequency domain. In order to correlate the signal characterization parameters with surface finish, multiple regression, and artificial neural network predictive models were analyzed. The original contribution of this study was to determine the optimum multi-sensor data system configuration, signal feature extraction method, and predictive modeling technique in terms of predictive power, reliability, and processing times for the real-time quality control of surface finish using only information from the sensors without off-line parameters.

## 2. Experimental Design and Methodology

**Figure 1:**
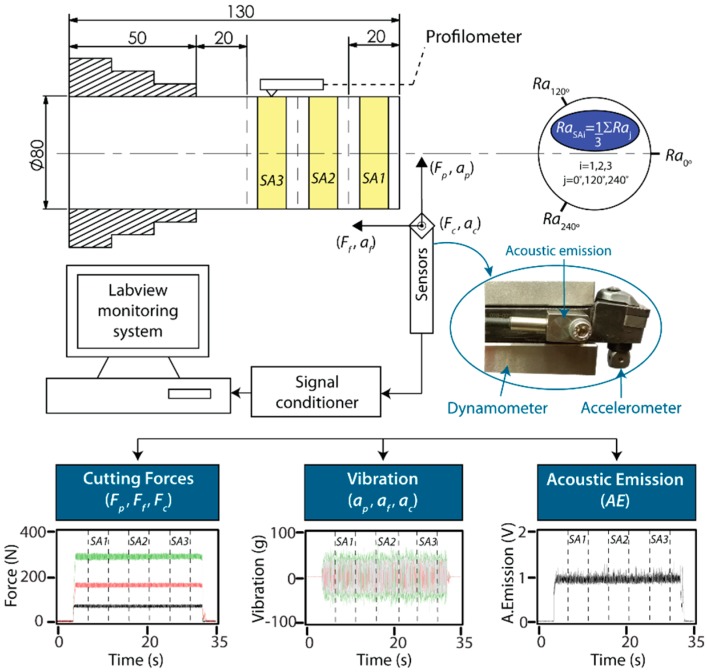
Experimental setup.

The machining trials were performed on a numerical control lathe (Goratu G Crono4S). Titanium carbonitride Ti(C,N) cutting inserts for finishing operations (0.4 mm corner radius) were used. Each workpiece was machined on a new cutting edge to avoid variability in cutting edge wear. The workpiece material was AISI 1045 steel, 80 mm in diameter and 130 mm in length, with 80 mm in cantilever (Figure 1). The experimental design was based on a factorial design with three factors at different levels: feed (*f*) six levels (0.08, 0.11, 0.14, 0.17, 0.20, 0.23 mm/rev), cutting speed (*v*) five levels (250, 275, 300, 325, 350 m/min), and cutting depth (*d*) four levels (0.5, 0.8, 1.1, 1.4 mm), with a total of 120 trial combinations. The machined length of each workpiece was subdivided into three 20 mm sampling areas (Figure 1), and signals were registered independently in each sampling area (*SA*1, *SA*2, *SA*3), with a total of 360 monitoring trials.

The parameter selected for the characterization of surface finish was the mean deviation of the assessed profile (*Ra*), as measured with a Talysurf Intra 50 profilometer. The cut-off (*λ_c_*) was 0.8 mm with an evaluation length (*l_t_*) of 4 mm. The arithmetic mean roughness value (*Ra*) was calculated for each sampling area (*Ra_SA_*_1_, *Ra_SA_*_2_, *Ra_SA_*_3_) as the average of the three equidistant measures in 120° rotation (Figure 1): $0^{{}^{\circ}}(Ra_{0^{{}^{\circ}}}),$ $120^{{}^{\circ}}(Ra_{120^{{}^{\circ}}})$, and $240^{{}^{\circ}}(Ra_{240^{{}^{\circ}}})$. The roughness measurements obtained for each sampling area were correlated to the signals registered in the machining trials.

The multi-sensor data system was designed in the LabVIEW virtual platform to process simultaneously cutting force (*F_p_*, *F_f_*, *F_c_*), mechanical vibration (*a_p_*, *a_f_*, *a_c_*), and acoustic emission (*AE*) signals with a dynamometer Kistler 9021, a triaxial accelerometer Kistler 8763B500BB, and a piezotron acoustic emission sensor 8152B111, respectively (Figure 1). In order to obtain adequate signal resolution, signals were captured with a sample frequency (*f_s_*) 5 times higher than the maximum frequency range of each sensor. The mechanical vibration signal was sampled with a data acquisition card NI PCI 6110 using a sample frequency *f_s_* = 50 kSamples/s, whereas cutting forces and acoustic emission (in RMS mode) were jointly sampled with a card NI PCI 6133 with a sample frequency *f_s_* = 5 kSamples/s.

**Figure 2:**
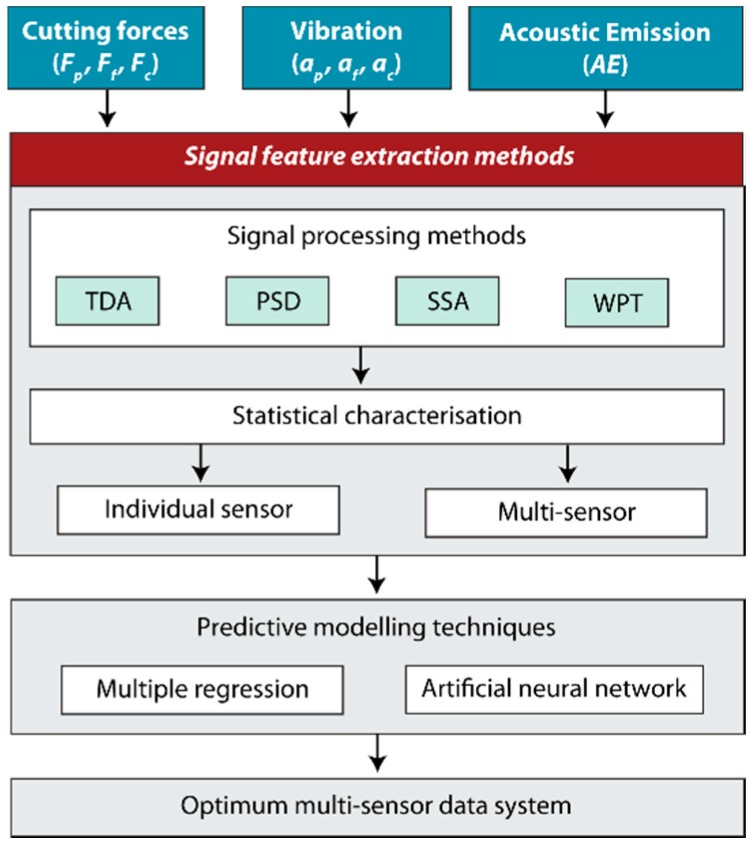
Methodology.

The methodology in this study involved the analysis of captured signals (*F_p_*, *F_f_*, *F_c_*, *a_p_*, *a_f_*, *a_c_*, *AE*) using the four signal feature extraction methods shown in Figure 2. The processed signals with TDA, PSD, SSA, and WPT methods were characterized by statistical and non-statistical parameters (Table 1). The signal feature extraction methods were evaluated in terms of each individual sensor, and multi-sensor. To correlate surface roughness with the signal characterization parameters, multiple regression and the artificial neural network were used as predictive modeling techniques. Finally, an optimum multi-sensor data fusion system was developed for real-time surface quality control.

Multiple regression and artificial neural network predictive models were evaluated in four ways: (1) the goodness of fit to experimental data by the adjusted determination coefficient $R_{adj}^{2}$; (2) the predictive power by the mean relative error ${\bar{e}}_{r}$ (Equation (1)) in the prediction of the experimental validation data, and the variability of ${\bar{e}}_{r}$ by the standard deviation associated to the mean value $\sigma_{{\bar{e}}_{r}}$; (3) the reliability in the prediction by the $R_{p}^{0.25}$ coefficient [53], and the percentage in the distribution error; and (4) the correlation of the data estimated by the predictive models versus the experimental validation data (*R*). All of the models under analysis reached the minimum requirement of a mean relative prediction error of ${\bar{e}}_{r}\leq 25\%$, and reliability of $R_{p}^{0.25}$ ≥ 75% [53]. Of the 360 experimental data obtained, 75% were used for building the models, and the remaining 25.0% were randomly selected for model validation. The multiple regression predictive models were adjusted stepwise to include only the significant (*p*-value < 0.05) characterization parameters (Table 1). All regression models were diagnosed by analyzing atypical values, multicollinearity, independence, and normality of the residuals, homoscedasticity, and contrasts and hypothesis tests.

(1) $${\bar{e}}_{r}\left(\%\right)=\frac{1}{n}\overset{n}{\underset{i=1}{\sum }}\left|\frac{\left(Ra_{i}^{exp}-Ra_{i}^{pred}\right)}{Ra_{i}^{exp}}\right|100$$

The design of the neural network was performed using a *feedforward* network structure with back-propagation training methodology. To determine the optimum network configuration, several training and transference functions widely used for the monitoring of machining processes were tested. As with the regression models, network validation was undertaken independently, with 25.0% of all the trials.

## 3. Results

### 3.1. Time Direct Analysis

**Figure 3:**
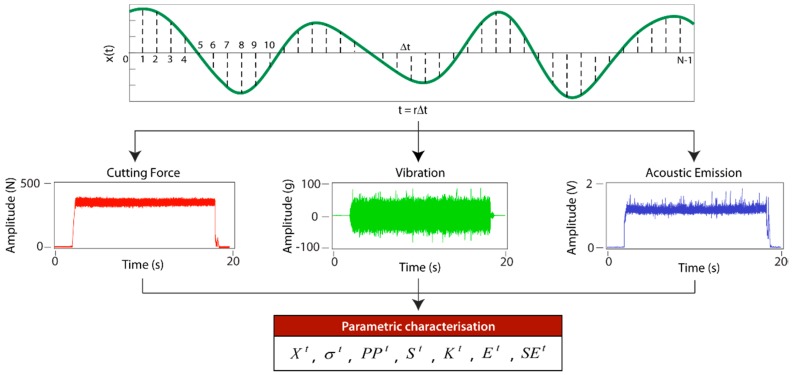
Time direct analysis signal processing method.

The TDA method directly analyses the signal registered by the sensor in the time domain, with no transformation or decomposition, for fast processing at a low analytical-computational cost. This method is based on signal definition as an amplitude-time function x(t) discretized by the succession $\left[x_{i}\right]$, with i=0,1,2,…,N−1, where *N* is the total number of points in the sample (Figure 3). The term *N* depends on sampling frequency (*f_s_*) and sampling time (*t*), which has a direct impact on processing times. Thus, cutting force (*F_p_*, *F_f_*, *F_c_*) and acoustic emission (*AE*) signals, both with $f_{s}=5\;\mathrm{kHz}$, required shorter processing times than vibration signals (*a_p_*, *a_f_*, *a_c_*) with $f_{s}=50\;\mathrm{kHz}$. The TDA method performs signal feature extraction using parametric characterization of the original signal captured by the sensor, defined in the time domain. For parametric characterization, statistical measurements (arithmetic mean, standard deviation variance, kurtosis Shannon entropy, etc.) or non-statistical measurements (energy, maximum and minimum peak amplitude, etc.) can be used [50]. The efficiency of the TDA method depends on the type of signal to be processed, and the information to be extracted.

The signal feature extraction of cutting forces, vibration and acoustic emission with the TDA method, involved direct parametric signal characterization using the parameters outlined in Table 1. In order to correlate the signal information with surface finish, the variables were related using a multiple regression predictive model with higher $R_{adj}^{2}$, lower mean relative error ${\bar{e}}_{r}$, and higher reliability $R_{p}^{0.25}$. For the optimum characterization of the triaxial signal sensors (dynamometer and accelerometer), the signals were analyzed independently for each component, and the fusion of the three components. This methodology identified correlations between components from each sensor to avoid information overloading of the predictive model that would undermine the fit.

**Figure 4:**
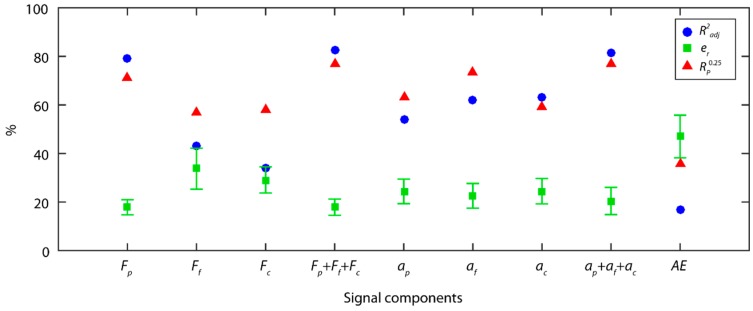
Signal analysis results with the TDA method.

The results obtained for cutting force signals with the TDA method (Figure 4) showed that the back force *F_p_* was the cutting force component most correlated to surface roughness (*Ra*), and provided the best results in all of the indices $(R_{adj}^{2}$ = 79.4%, ${\bar{e}}_{r}$ = 17.8 ± 3.1%, $R_{p}^{0.25}$ = 71.1%). The back force *F_p_* is responsible for tool–workpiece contact stability, and flexing of the workpiece machined in cantilever, given that this force was perpendicular to the axis of rotation of the workpiece. These results underscore the impact of this component (*F_p_*) on surface finish, due to the influence of the tool–workpiece interaction, and the dynamic behavior of the rotated workpiece. The feed force *F_f_*, and tangential force *F_c_* showed weaker correlations to roughness, with an $R_{adj}^{2}$ percentages of 43.4% and 34.0%, respectively. The model combining the three force components $\left(F_{p}+F_{f}+F_{c}\right)$, slightly improved the results of the *F_p_* force model, increasing reliability to 76.7%, and improving the data fit to 82.5%, but with no improvement in predictive power with an ${\bar{e}}_{r}$ of 17.9 ± 3.4%. This corroborated that the back force *F_p_* explained a larger percentage of the variability in the experimental data, indicating it was the component with the greatest impact on roughness (*Ra*). The feed force *F_f_* and tangential force *F_c_* complemented the information provided by the back force *F_p_*, but the improvement in the fused model was relatively small.

With the TDA method, the individual analysis of vibration signal models $\left(a_{i}\right)$ exhibited a moderate fit to data, with an $R_{adj}^{2}$ below 63% for all of the components, and an ${\bar{e}}_{r}$ close to the critical value of 25% but never surpassing it. The three vibration components provided similar percentages of information, with the feed vibration *a_f_* ($R_{adj}^{2}$ = 62.2%) and radial vibration *a_p_* ($R_{adj}^{2}$ = 62.9%) explaining most variability of the experimental data, and to a lesser extent the *a_p_* percentage (54.1%). The fused vibration model $\left(a_{p}+a_{f}+a_{c}\right)$ significantly improved model prediction, both in terms of the fit to data (81.6%), predictive power (20.5 ± 5.6%), and reliability (76.7%). This implied that the three vibration components $\left(a_{p},a_{f},a_{c}\right)$ had a similar impact on surface finish, with a lower correlation among them than for the cutting force components.

The acoustic emission (*AE*) model obtained very poor results in all of the indicators analyzed, having little impact on the parameter *Ra*. This model explained only 17.1% of the variability of the experimental data, which indicated a very poor correlation to roughness (*Ra*). This implies information extracted for the *AE* signal and processed with the TDA method did not permit the correlation between this signal and surface roughness.

**Figure 5:**
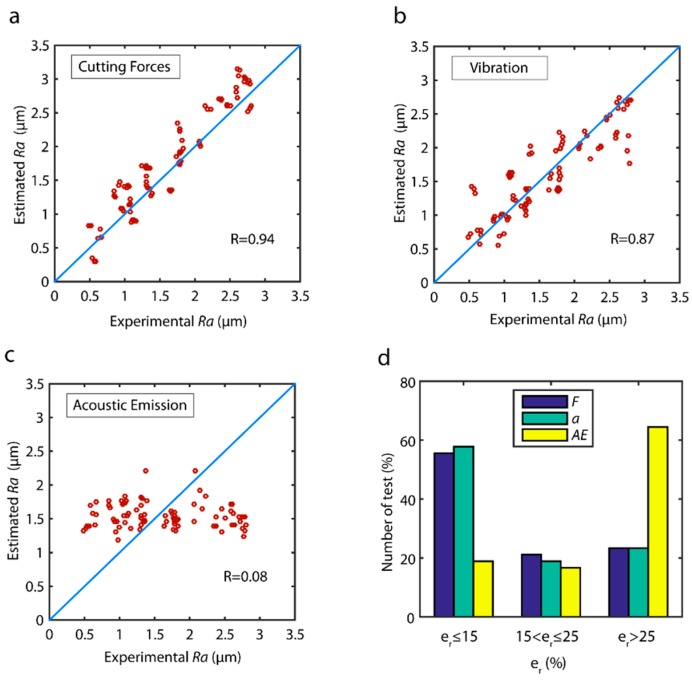
Estimated values versus experimental validation values for the parameter *Ra* with the TDA method: (**a**) cutting forces, (**b**) vibration, and (**c**) acoustic emission. (**d**) Prediction reliability with the TDA method.

The correlations of the estimated data versus the validation data for the best model obtained with each sensor are shown in Figure 5. Cutting force signals exhibited the best behavior (Figure 5a) with a correlation R=0.94, and uniform behavior in all of the data ranges. The model tended to slightly overestimate, given that most of the estimated values were higher than the experimental validation values. As shown in Figure 5b, in the vibration model greater dispersion was observed in all of the value ranges, which weakened the correlation of R=0.87 in comparison to cutting forces. As for acoustic emission (Figure 5c), a very poor correlation R=0.08 was obtained, indicating a very poor correlation between this signal *AE* and surface finish with the TDA method. In relation to model reliability in terms of the distribution error of the validation data (Figure 5d), a similar behavior was observed between the cutting force model and the vibration model. In both models, from 56.0% to 58.0% of the validation trials were optimally estimated with an $e_{r}\leq 15\%$, and 76.7% of trials were satisfactorily predicted with error $e_{r}\leq 25\%$. The poor behavior observed with acoustic emission signal generated a model with 75% of estimated data out of the acceptable range.

### 3.2. Power Spectral Density

The PSD is a real positive function to calculate the distribution of the power of an original signal *x*(*t*) along the entire frequency range registered by the signal. The PSD can be calculated according to Wiener–Khintchin’s theorem as the Fourier transform of the autocorrelation function (Equation (2)).

(2) $$S_{x}(f)=\int_{-T}^{T}R_{x}(\tau )e^{-j2\pi ft}dt\;\mathrm{where}\;R_{x}(\tau )=E\{x(t)x^{*}\left(t+\tau \right)\}$$

**Figure 6:**
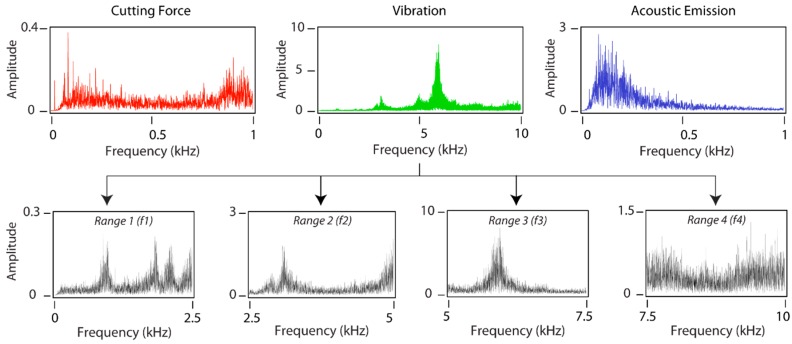
PSD signal processing method with four frequency ranges.

As mentioned in Section 2, the frequency ranges of the sensors were different: for cutting force and acoustic emission signals maximum frequency was ~1 kHz, whereas the maximum frequency for vibration was ~10 kHz. The broad bandwidth of the vibration signal entailed certain frequency ranges with significant information failed to be adequately characterized. Thus, the frequency analysis of the vibration signal was undertaken using two methods: a complete analysis of the entire bandwidth and a fractioned analysis by discretizing the bandwidth into four independent frequency ranges as shown in Figure 6.

**Figure 7:**
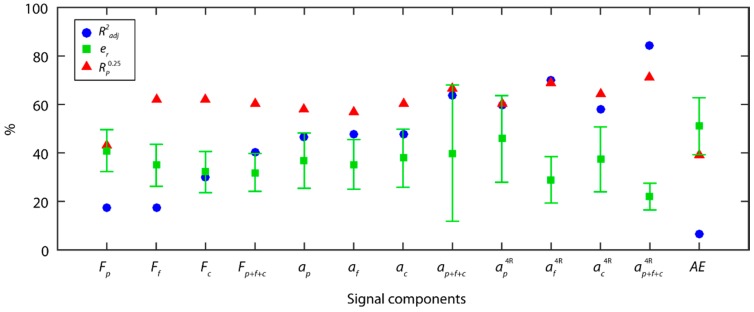
Signal analysis results with the PSD method.

Following signal feature extraction and the building of the predictive models, Figure 7 shows the results obtained with the PSD method for each signal. For cutting force signals, neither the models built for each force component (*F_p_*, *F_f_*, *F_c_*), nor the models of the fused components $\left(F_{p}+F_{f}+F_{c}\right)$, obtained satisfactory results, and neither reached the minimum criteria for a model to be considered acceptable. Vibration signal analysis in four frequency ranges (4R) improved the results obtained as compared to signal analysis in one single frequency range, but the mean relative errors and reliability were deficient. The feed vibration *a_f_* was the most correlated to surface roughness with a $R_{adj}^{2}$ of 70.1 %, with a lower correlation for the back vibration *a_p_*, and tangential vibration *a_c_* components of 46.4% and 47.5% of $R_{adj}^{2}$, respectively. The fused vibration model $\left(a_{p}+a_{f}+a_{c}\right)$ obtained the best results $(R_{adj}^{2}=84.0\%,$ ${\bar{e}}_{r}=22.0\pm 5.5\%$, $R_{p}^{0.25}=71.1\%),$ underscoring it was the best predictive model of those analyzed. Similar to TDA method, the acoustic emission signals analyzed with the PSD method also obtained poor results, with a low correlation between this signal and surface finish.

**Figure 8:**
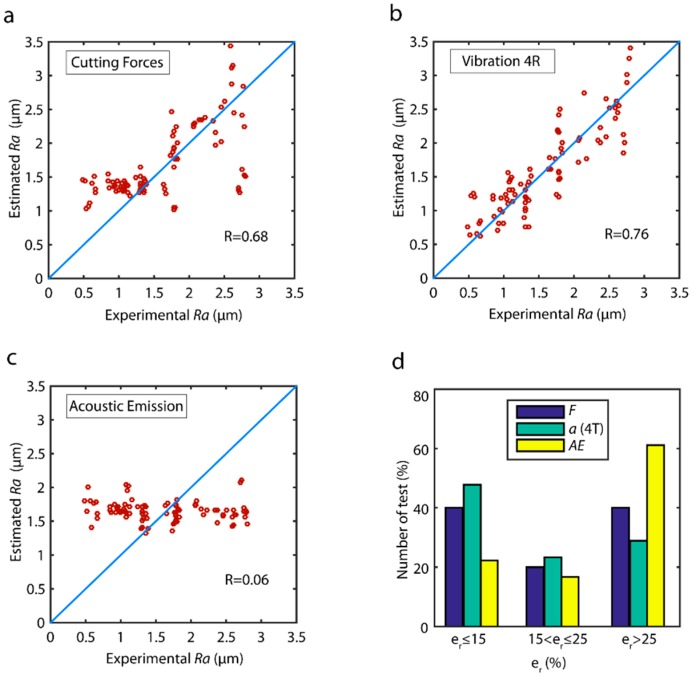
Estimated values versus experimental validation values of the parameter *Ra* for the PSD method: (**a**) cutting forces, (**b**) vibration (4R), and (**c**) acoustic emission. (**d**) Prediction reliability with the PSD method.

With reference to estimated validation data (Figure 8), the best cutting force model $\left(F_{p}+F_{f}+F_{c}\right)$ obtained a low correlation of R=0.68 (Figure 8a), with inaccurate estimates in high and low roughness values, and a slightly better behavior in mid-range values. The fused vibration model $\left(a_{p}+a_{f}+a_{c}\right)$ with four frequency ranges (4R) (Figure 8b) obtained the highest correlation (*R* = 0.76), with uniform estimated data in all of the ranges of surface finish (Figure 8b). The acoustic emission signal was not correlated in anyway (R=0.06) with very deficient results being obtained.

The analysis of model reliability in terms of the distribution error in the prediction of the validation data (Figure 8d), revealed the best model was fused vibration signals $\left(a_{p}+a_{f}+a_{c}\right)$ in four frequency ranges with an $R_{p}^{0.25}$ of 71.1%, followed by fused cutting forces model $\left(F_{p}+F_{f}+F_{c}\right)$ with an $R_{p}^{0.25}$ of 60.0%, and acoustic emission with very poor results ($R_{p}^{0.25}$ = 38.9%).

The analysis revealed the PSD method failed to provide adequate signal feature extraction for the prediction of surface roughness, given that none of the models analyzed reached a prediction reliability of 75%.

### 3.3. Singular Spectrum Analysis

The SSA method is a non-parametric time series analysis technique, based on statistical multivariability, multivariante geometry, and dynamic signal processing systems. The SSA method decomposes a signal into independent time series (with defined frequency ranges) referred to as principal components (PC_i_). The SSA method builds a Hankel matrix termed the trajectory matrix X, calculated through a sliding windows (*L*) applied to the succession of data $\left[x_{i}\right]$ from the original signal. The next step is singular value decomposition (SVD) of the trajectory matrix by decomposing the X matrix into a series of elementary matrixes $X_{i}$ obtained by calculating the eigenvalues and eigenvectors of the matrix $S=XX^{T}.$ Finally, the principal components are obtained from the reconstruction of the elementary matrixes $X_{i}$ [50]. The number of principal components obtained with the SSA method depends directly on the parameter *L*, which is a significant factor conditioning the results. To determine the principal components containing information of the original signal, it is standard practice to show in descending order the weight of each principal component (or eigenvalue) on a graph commonly referred to as Singular Spectrum (*SS*), which give its name to the SSA method itself.

**Figure 9:**
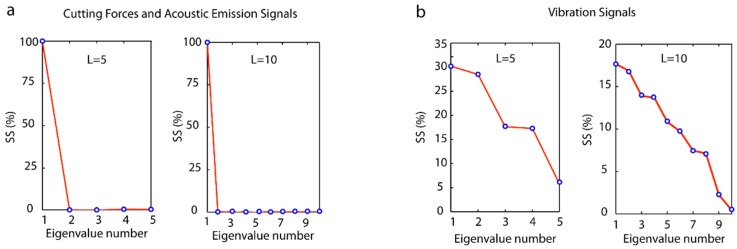
Signal analysis with the SSA method using two window lengths *L* = 5 and *L* = 10: (**a**) cutting forces and acoustic emission, and (**b**) vibration.

The application of *SS* to cutting force, vibration, and acoustic emission signals for the window lengths *L* = 5 and *L* = 10, showed significant differences (Figure 9). A similar behavior was observed in cutting forces and acoustic emission (Figure 9a), where the eigenvalue associated to the first principal component (PC_1_) contained approximately 100% of the weight of the total signal, and the remaining principal component values obtained were almost negligent. The application of the SSA method to vibration signals exhibited a different behavior (Figure 9b) with non-zero eigenvalues, resulting in principal components with different levels of information.

**Figure 10:**
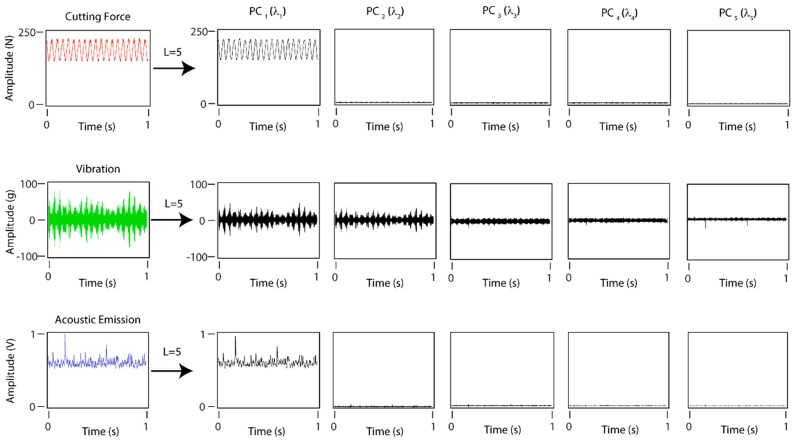
Principal components obtained with the WPT method for cutting forces, vibration, and acoustic emission, and a window length of *L* = 5.

Figure 10 shows the principal components obtained with the SSA method for a window length of *L* = 5. Cutting forces and acoustic emission only contained information in the first principal component (PC_1_), the remaining components being negligible, which corroborated the *SS* results (Figure 9). The first principal component (PC_1_) was the original signal, thus the results obtained for the signals with the SSA method were equivalent to those obtained with the TDA method. This phenomenon occurred in signals with a characteristic function *x*(*t*) = *A*sin*t* + *b* where the factor *b* represented signal amplitude, and the term *A*sin*t* the signal oscillation. With SSA decomposition, this amplitude and the oscillation range were contained in the first principal component, and the remaining components provided little or negligible additional information. Moreover, the vibration signal obtained significant information from the original signal in the five principal components as these signals had a value of *b* ≈ 0. The selection of the vibration signal configuration parameters for the SSA method was determined on the basis of the results obtained by García and Núñez [50].

**Figure 11:**
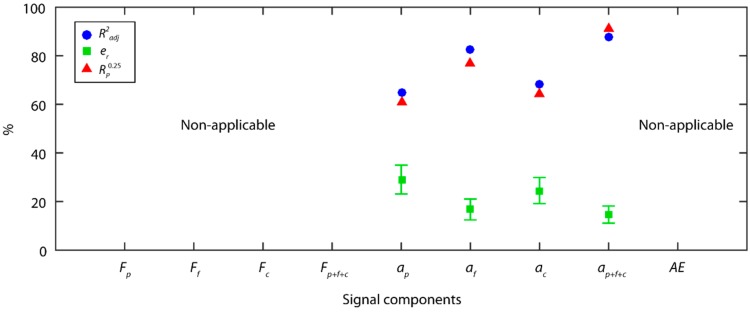
Signal analysis results with the SSA method.

The individual analysis of the vibration signals with the SSA method (Figure 11) showed the feed vibration *a_f_* explained most of the variability in the experimental data with a fit of data of 82.8%, a predictive power of 16.7 ± 4.3%, and 76.7% of reliability. The radial vibration *a_p_* and tangential vibration *a_c_* components explained less variability (65.1% and 68.4%, respectively) with high relative errors (29.0% and 24.5%, respectively) and low reliabilities (61.1% and 64.4%, respectively) that failed to reach acceptable levels. In contrast, the combination of the vibration components $\left(a_{p}+a_{f}+a_{c}\right)$ improved the model in all of the evaluation indices of the individual analyses, with a $R_{adj}^{2}$ of 87.8%, sharp fall in the relative error ${\bar{e}}_{r}$ reaching a value of 14.6%, and a significant increase in reliability to 91.1%. These results revealed that feed vibration *a_f_* was the component with the greatest impact on surface finish (*Ra*), and that the radial *a_p_* and tangential *a_c_* vibration complemented the information provided by *a_f_*.

**Figure 12:**
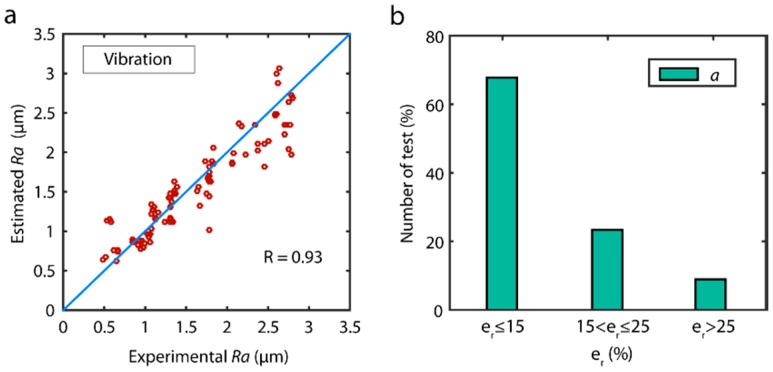
(**a**) Estimated data versus experimental validation data of the parameter *Ra*. (**b**) Reliability of predictive models.

The prediction of the validation data versus the experimental values for the best vibration model obtain with SSA method $\left(a_{p}+a_{f}+a_{c}\right)$ is shown in Figure 12a. The results showed the model had a good predictive power with a high correlation (R=0.93), without any significant bias, except for a slight underestimation in the prediction of *Ra* values above 2.5 μm. The analysis of model reliability in terms of the distribution error in the prediction of the validation data showed the model had very good reliability (Figure 12b), with 67.8% optimum estimation of the data $\left(e_{r}\leq 15\%\right)$, and 91.1% acceptable predictions $\left(e_{r}\leq 25\%\right)$.

### 3.4. Wavelet Packet Transform

The WPT method decomposes a signal into scaled and shifted series (packets) of a prototype function referred to as the mother wavelet, which is characterized on a time-frequency scale. The method applies a pyramidal algorithm, where the original signal is successively split into approximation *A_j_* (low frequencies) and detail *D_j_* (high frequency) signals until the desired wavelet decomposition level is achieved. The approximation and detail signals are calculated by the coefficients described in Equations (3) and (4),

(3) $$A_{j}\;(k)=\underset{n}{\sum }h(n-2k)c_{j-1}(n)$$

(4) $$D_{j}\;(k)=\underset{n}{\sum }g(n-2k)c_{j-1}(n)$$

where $A_{j\;}(k)$ and $D_{j\;}(k)$ are approximation and detail coefficients, *j* is the number of transformation levels with j=1, 2, …; *k* is the number of scaled and wavelet coefficients with $k=1,\;2,\;\ldots ,\;Nx2^{-j}$, where *N* is the total number of samples of the original signal; *h* and *g* are low-pass and high-pass coefficients of the scaled function and wavelet function, respectively, based on a chosen mother wavelet; and *n* is the filter length.

For the satisfactory application of the WPT, three fundamental factors should be borne in mind: (1) selection of the appropriate mother wavelet for each specific type of signal; (2) to determine the number of decomposition levels (*L_d_*) needed to divide the signal into effective frequency ranges; and (3) selection of the information packets of significance to the parameter under analysis. The optimum selection of the configuration parameters applied in the WPT method to cutting force and vibration signals for the monitoring of surface finish was determined by García and Núñez in two previous studies [52,53], respectively, where the mother wavelet *bior4.4* with three decomposition levels provided the best results for vibration signals, and the mother wavelet *db06* with four decomposition levels provided the best results for cutting forces. As there are no studies published in the literature determining the optimum configuration for acoustic emission signals, this study replicated the methodology employed by García y Núñez, establishing the best configuration for mother wavelet *coifflet4.4* (*coif4.4*), and a number of decomposition levels *L_d_* = 5.

**Figure 13:**
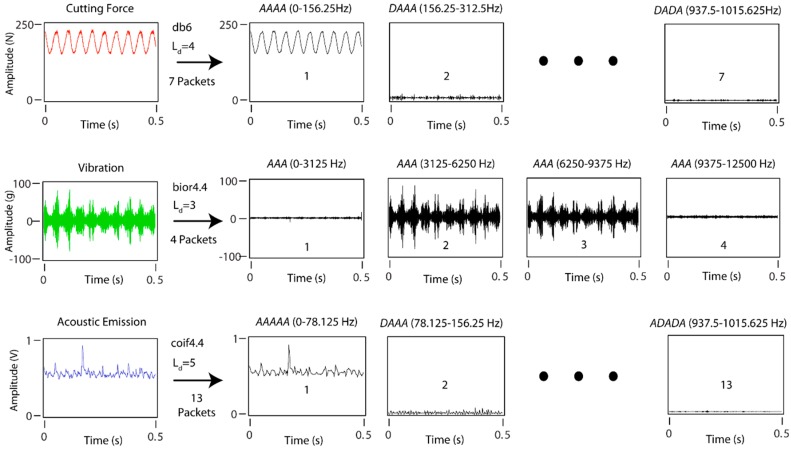
WPT method applied to cutting force, vibration, and acoustic emission signals.

The sampling frequency (*f_s_*), and decomposition level (*L_d_*) determined the frequency ranges of the original signal (Figure 13). For cutting force signals, seven frequency ranges of 156.25 Hz were obtained, for vibration signals four frequency ranges of 3125 Hz, and for acoustic emission 13 frequency ranges of 78.125 Hz (Figure 13).

**Figure 14:**
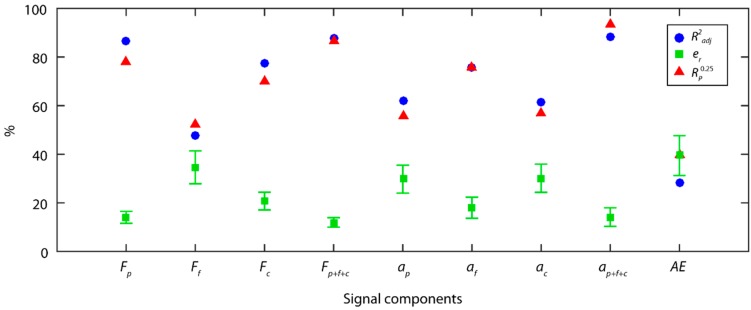
Signal analysis results with the WPT method.

As shown in Figure 14, in the individual analysis of cutting forces, the back force *F_p_* was the principal information source explaining most of the variability in the experimental data, with a data fit of 86.4% and 14.1 ± 2.5% of predictive power. As with the TDA method, the back force *F_p_* was the cutting force component having the greatest impact on surface finish, with better prediction indices than in the TDA method. Nevertheless, in this case the tangential force *F_c_* also obtained a good fit to the experimental data with an $R_{adj}^{2}$ of 77.7%, and an ${\bar{e}}_{r}$ of 20.7 ± 3.6%. As shown in the analysis, the WPT method only analyzed time series in effective frequency ranges with relevant information, excluding series that masked the original signal, and impeded adequate signal feature extraction in methods such as the TDA. This enhanced the analysis of cutting force signals, eliminated noise from the signal, with the tangential force *F_c_* exhibiting a greater influence on roughness.

The fusion of the three orthogonal cutting force components $\left(F_{p}+F_{f}+F_{c}\right)$ hardly improved the fit of the model with an $R_{adj}^{2}$ of 88.0%, indicating a strong correlation among the cutting force components. In contrast, the fused model $\left(F_{p}+F_{f}+F_{c}\right)$ improved the predictive power with an ${\bar{e}}_{r}$ of 11.9 ± 1.9%, and an 86.7% of reliability. These results revealed that the back force *F_p_*, and tangential *F_c_* were had the greatest influence on surface finish (*Ra*), and the feed force *F_f_* complemented the information, improving the predictive power with the fusion of the cutting force components.

The results obtained for the vibration signals were similar to those obtained with the SSA method. In the individual analysis of the vibration components (*a_p_*, *a_f_*, *a_c_*), once again the feed vibration *a_f_* provided most of the information, with a data fit of 75.7%, a predictive power of 18.0 ± 4.3%, and a reliability of 75.6%. The results for the back *a_p_* $\left(R_{adj}^{2}=62.0\%\right)$ and tangential *a_c_* $\left(R_{adj}^{2}=61.2\%\right)$ components were poorer than those obtained for *a_f_* with high relative errors and low reliabilities that failed to reach minimum acceptable levels. The combination of the three vibration components $\left(a_{p}+a_{f}+a_{c}\right)$ improved the model in all of the evaluation indices $(R_{adj}^{2}=88.5\%,$ ${\bar{e}}_{r}=14.2\pm 3.8\%$, $R_{p}^{0.25}=93.3\%)$ The acoustic emission signal (*AE*) failed to obtain good results with the WPT method, with all of the indicators of predictive power being deficient.

**Figure 15:**
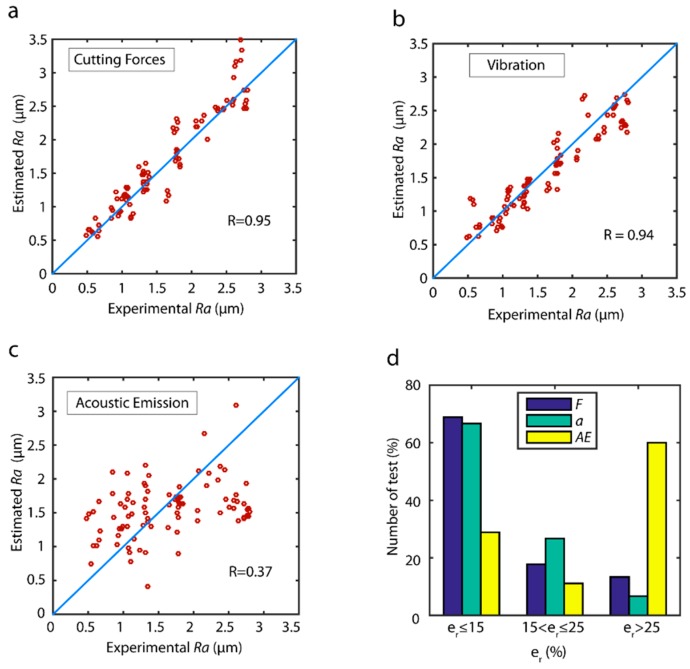
Estimated data versus experimental validation data for the parameter *Ra*: (**a**) cutting forces, (**b**) vibration, and (**c**) acoustic emission. (**d**) Reliability of predictive models.

The correlation between estimated data versus the validation data for the model obtained for each sensor is shown in Figure 15. The cutting force and vibration models obtained the best results with very high *R* correlations of 0.95 and 0.94, respectively. Though the data distribution in both models was fairly uniform in all of the ranges of surface finish (*Ra*), a slight overestimation was observed in all of the data ranges in the cutting force model (Figure 15a), which tended to increase at higher roughness values (2.6 µm ≤ *Ra* ≤ 3.0 µm). As for acoustic emission (Figure 15c), the correlation obtained was very deficient (R=0.37). The analysis of model reliability (Figure 15d) revealed a similar behavior, with cutting force and vibration models obtaining the best results. Both models obtained similar results in the optimum prediction range, where 67.0–69.0% of the validation trials had an estimated error $e_{r}\leq 15\%$; however, in the interval of acceptable predictions, the vibration model obtained the best results, with an error $e_{r}\leq 25\%$ in 93.3% of the validation data, compared to 86.7% for the cutting force model. The poor behavior obtained with the acoustic emission signal produced a model where 60.0% of estimated data was outside the acceptable range.

### 3.5. Comparison of Methods

**Figure 16:**
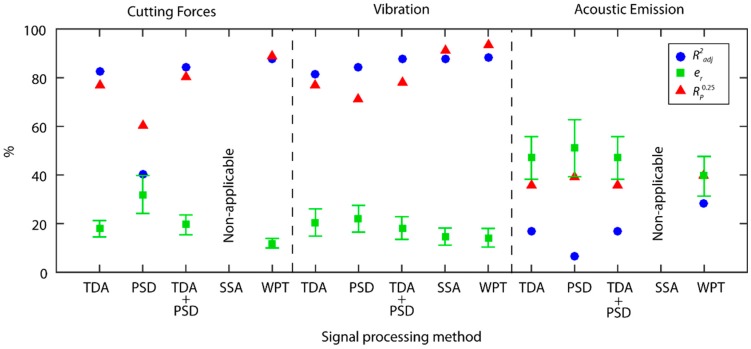
Comparison of the predictive results according to processing method and signal type.

**Figure 17:**
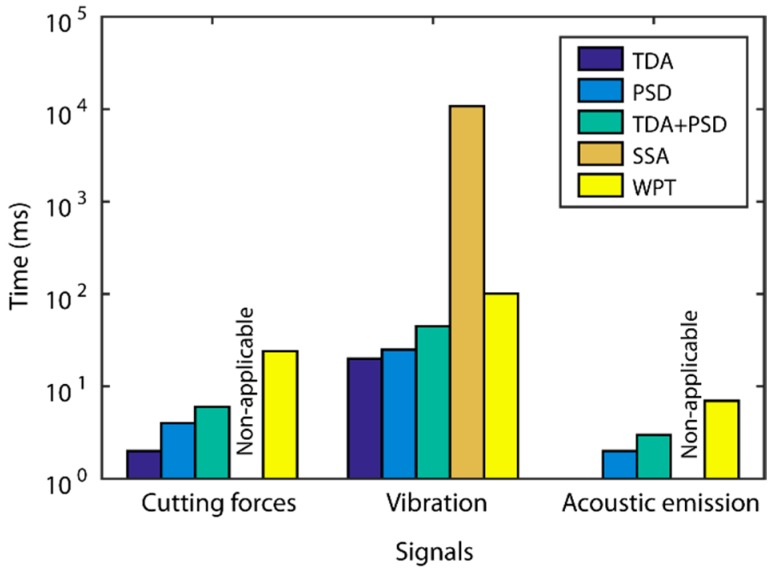
Response times obtained for the TDA, PSD, TDA + PSD, SSA, and WPT processing methods.

A comparison of the best predictive models obtained in the previous section, classified according to processing method and the type of signal under analysis, is shown in Figure 16. In order to draw a more accurate comparison of the processing methods in a single domain (TDA and PSD) versus methods of analysis in the time-frequency domain (SSA and WPT), the predictive models obtained with the fusion of the TDA and PSD methods were compared. To evaluate the computational cost of each method, the monitoring system response time in the processing of a second signal was analyzed (Figure 17).

In the analysis of cutting forces, the WPT method presented the best prediction results with an ${\bar{e}}_{r}$ of 12.0 ± 2.0%, a reliability of 88.7%, and a 24 ms response time. As shown in Figure 16, the results obtained with the other methods fell far short of the method WPT. It should be noted that the characterization of cutting force signals in the frequency domain presented the worst results, and the combination with the TDA method worsened the predictive power ${\bar{e}}_{r}$ of the TDA method.

In the analysis of the vibration signals, the SSA and WPT methods presented the best prediction results with an ${\bar{e}}_{r}$ of 14.6 ± 3.5% and 14.2 ± 3.8%, and reliability of 91.1% and 93.3%, respectively. In comparison, the prediction results obtained with the other methods fell far short of the SSA and WPT methods. In this case, PSD analysis provided complementary information to the TDA analysis, which improved the prediction and reliability results. For the vibration signals, system response times increased significantly as sampling frequency was five times greater than in cutting force signals. In spite of this increase, the processing time of the WPT method was sufficiently low for real-time monitoring (101 ms), but the SSA method presented a very long response time (10,750 ms), which discarded it as feasible for the real-time prediction of surface finish.

Acoustic emission failed to provide good results in all of the methods under analysis, with a slight improvement in the WPT method, but failing to obtain satisfactory results. The lowest computational costs were for acoustic emission in comparison to the other signals, owing to the smaller amount of information processed in one single signal.

## 4. Multi-Sensor Data Fusion Analysis

### 4.1. Comparative Analysis of Sensor Fusion

Having determined the behavior of the processing methods individually for each signal, the next step was to analyze the multi-sensor fusion data by building a fused model of each processing method, using the significant characterization parameters obtained in each individual analysis. It should be borne in mind that the acoustic emission signal provided poor prediction results in each individual analysis, which indicated the *AE* signal contained no information correlated to surface finish. Thus, two types of analysis of the fusion of sensors were performed: the fusion of all the sensors, and the fusion of all the sensors except the acoustic emission signal. It should be noted that the SSA method can only be applied efficaciously to vibration signals as explained previously in Section 3.3. Nevertheless, the SSA method was applied to all of the signals in order to compare the fusion of sensors, taking into account that cutting force and acoustic emission signals were equivalent to applying the TDA method.

**Figure 18:**
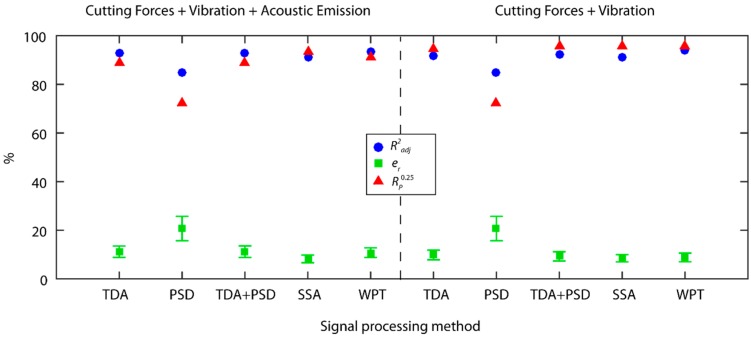
Comparison of the prediction results obtained with multi-sensor data fusion with and without the acoustic emission signal.

As shown in Figure 18, with exception of PSD analysis, the results obtained with the other methods and the fusion of three sensors provided predictive models with low relative errors ${\bar{e}}_{r}<12\%$, and high reliability over 88.0%, which improved the results obtained with the individual analysis of each sensor. The best result was obtained for the SSA method, with excellent predictive power ${\bar{e}}_{r}=8.2\pm 1.6\%$, and very high reliability of 93.3%. Similar results were obtained for the WPT method with a mean relative error of 10.8 ± 2.0% and a reliability of 91.1%.

When the *AE* signal was eliminated from multi-sensor data fusion, with the exception of the model of the PSD method, the predictive power of the other models improved, indicating the *AE* signal provided no significant information for the prediction of surface finish, or even negatively affected the prediction. The results for the fusion of cutting force and vibration signals were similar with hardly any considerable differences between them, with the exception of the PSD method. All of the methods exhibited an excellent predictive power, particularly the WPT and SSA methods that obtained an excellent mean relative error of 8.5 ± 1.5% and 8.8 ± 1.8%, respectively, with an optimum reliability of 95.5% in both. Even the TDA method and TDA+PSD fusion obtained excellent results with the fusion of sensors.

**Figure 19:**
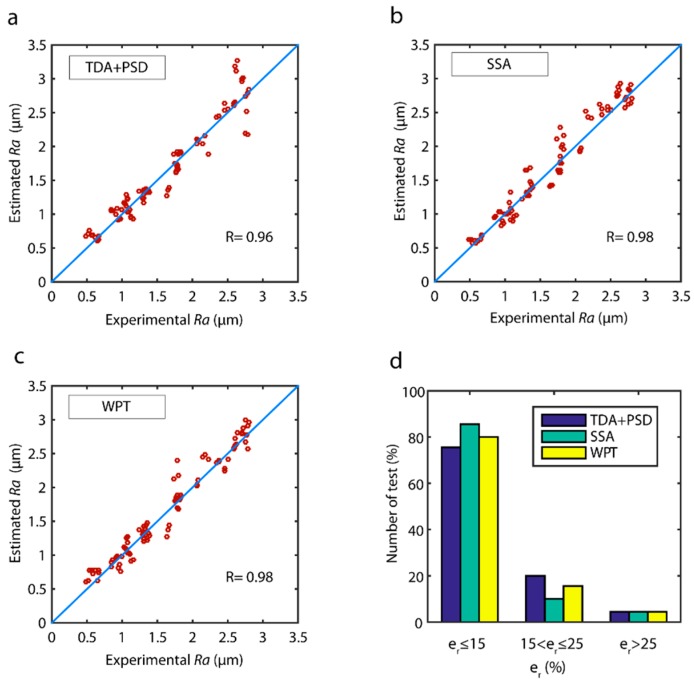
Estimated data versus experimental validation data for the parameter *Ra* with multi-sensor data fusion for the (**a**) TDA + PSD, (**b**) SSA, (**c**) WPT methods, and (**d**) model reliability.

The correlations between the estimated data and the validation data of the models obtained with the fusion of sensors are shown in Figure 19. The three models had very strong correlations (Figure 19), with the SSA (Figure 19b) and WPT (Figure 19b) methods exhibiting a uniform behavior in the entire range of experimental data. However, the TDA + PSD method underperformed in data prediction at the 2.5 ÷ 3.0 μm interval. The analysis of model reliability (Figure 19d), in the range of optimum predictions $(e_{r}\leq 15\%),$ showed the SSA model had the best performance with 85.5% of the data, followed by the WPT model with 80.0% of the data, and the TDA + PSD model with 75.5%. In the acceptable prediction range $\left(e_{r}\leq 25\%\right)$, excellent results were obtained for the three models with a 95.5% reliability in all three models.

Bearing in mind these data and the aforementioned computational costs, the WPT model with the fusion of cutting force and vibration sensors was the best option for the time-real prediction of surface quality in CNC automated machining processes. Moreover, the WPT method was applicable to all of the signals analyzed and enabled the determination of effective frequency ranges correlated to surface finish. The significant characterization parameters obtained with the WPT model and the corresponding frequency ranges, the sum of squares (type III), and the *p*-values are shown in Table 2. The most relevant information of cutting force signals was found at very low frequency ranges *AAAA* (0–156.25 Hz), with a small contribution from low *DAAA* (156.25–312.50 Hz), and very high *DADA* frequencies (937.50–1093.75 Hz), and negligible information from the other frequency ranges analyzed. The behavior of the vibration signals was entirely different with high frequency *DDA* (6250–9375 Hz) providing most of the relevant information, followed by the very high *ADA* frequency (9375–12,500 Hz), and no information provided by the other frequency ranges.

As for the level of information provided by the cutting forces, the mean of the back force $X_{F_{p}}^{AAAA},$ and Shannon entropy of the tangential force $SE_{F_{c}}^{AAAA}$ were the primary information sources. Both components (*F_p_* and *F_c_*) were responsible for the load on the tool in the direction perpendicular to the axis of rotation, and flexing of the workpiece at the cantilever. This aspect led to displacement and eccentric rotation of the workpiece, which altered the dynamic behavior and causes vibrations in workpiece-tool contact areas. The parameter $X_{F_{p}}^{AAAA}$ measured the static component of the back force *F_p_*, and $SE_{F_{c}}^{AAAA}$ measured the dynamic component of tangential force *F_c_*.

In relation to the vibration signal, the parameters measuring the dynamic behavior of the signal were the most significant, with the feed $\sigma_{a_{f}}^{DDA}$ and tangential $\sigma_{a_{c}}^{DDA}$ standard deviation being the principal sources of information, complemented by mean feed vibration $X_{a_{f}}^{ADA}$ and the entropy of the three components $SE_{a_{p}}^{DDA}$, $SE_{a_{f}}^{DDA}$, $SE_{a_{f}}^{ADA}$, $SE_{a_{c}}^{ADA}$. The feed component of the vibration signal provided most of the variables with the greatest impact on the prediction of surface roughness. Feed vibration *a_f_* was the vibration component most affecting surface roughness, and was directly correlated to the parameter *Ra*.

### 4.2. Comparative Analysis of Predictive Techniques

The ANN optimized for the prediction of surface finish had a feedforward structure with back-propagation training methodology. In order to obtain the optimum network configuration, the training and transference functions available in Matlab 2018 were analyzed using a pyramidal criterion [67] to determine the number of layers and neurons providing the best results. Similar to the multiple regression models, network validation was undertaken independently with 25.0% of the randomly selected experimental data. The input variables were restricted to significant signal characterization parameters obtained with the regression models to ensure the fit of the network was not weakened due to information overloading or correlations between variables. The optimum networks obtained for the TDA + PSD and SSA methods had a structure 13 × 6 × 2 × 1 and 10 × 3 × 1, respectively, with the *tansig* transference function and *trainlm* training. The network for the WPT method had a structure 12 × 3 × 1 with the *purelin* transfer function and *trainlm* training.

**Figure 20:**
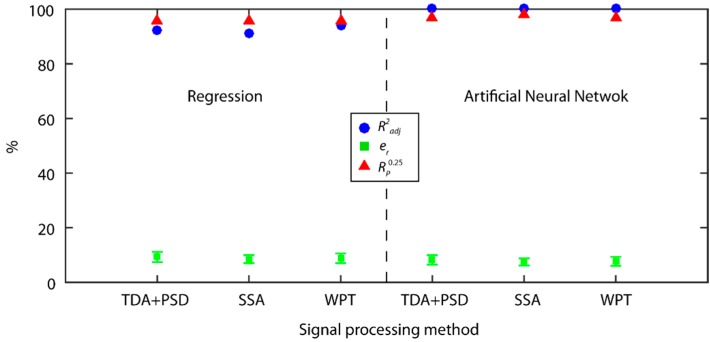
Prediction results for the MR and ANN models with multi-sensor data fusion of cutting force and vibration signals.

The results obtained for the prediction of surface finish with ANN and multiple regression are shown in Figure 20. The two prediction techniques analyzed obtained excellent prediction results with ${\bar{e}}_{r}$ below 10.0 ± 2.0%, and reliability over 95.0% for the three methods analyzed (TDA + PSD, SSA, and WPT). The ANN predictive modeling technique improved the ${\bar{e}}_{r}$ from 1.0% to 1.2 %, obtaining 8.2 ± 1.7% for TDA + PSD, 7.5 ± 1.3% for SSA, and 7.7 ± 1.6% for the WPT method. As for reliability, a 1.1% to 2.2% improvement was observed, obtaining 96.7% for the TDA + PSD, 97.8% for SSA, and 96.7% for the WPT method. Though the improvement in prediction and reliability may initially appear to be insignificant, it entailed a substantial increase in the performance of the predictive model, owing to the low prediction errors in both models, and the precision requirements of surface quality control systems.

**Figure 21:**
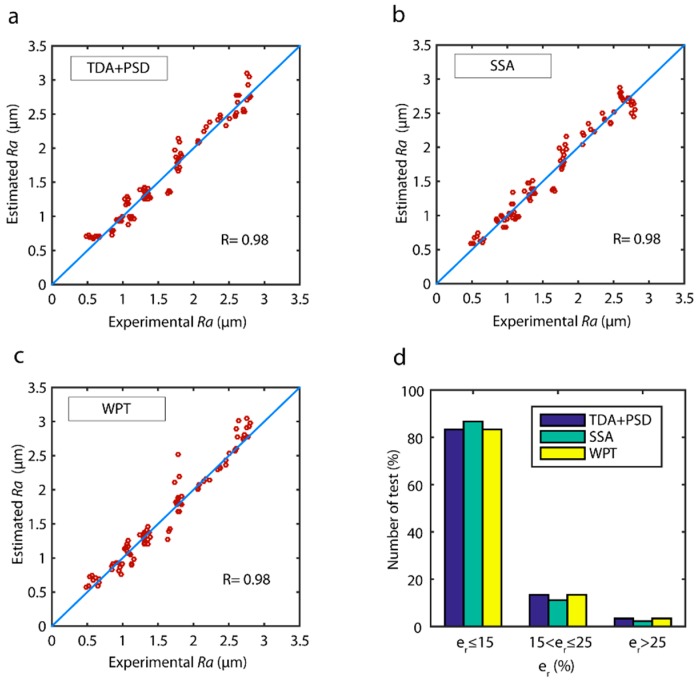
Correlation of estimated data versus validation experimental data obtained with ANN predictive model and multi-sensor data fusion: (**a**) TDA + PSD, (**b**) SSA, and (**c**) WPT methods. (**d**) Prediction reliability with the ANN model.

The correlation of estimated data versus experimental validation data (Figure 21) of the ANN models was very high, with excellent behavior in all of the value ranges. The reliability of the models (Figure 21d) was very similar, with the SSA method showing the best results with 86.6% of the predictive data in the optimum range $\left(e_{r}\leq 15\%\right)$ versus the TDA + PSD and WPT methods that reached a reliability of 83.3%. As for the range of acceptable predictions $\left(e_{r}\leq 25\%\right)$, the three models presented similar results with 97.8% for the SSA, and 96.7% for the TDA + PSD and WPT methods, indicating only from 2.2% to 3.3% of the data was predicted with an *e_r_* greater than 25.0%.

The best prediction and reliability results were obtained with the ANN model for the SSA and WPT methods, reaching excellent precision and reliability indices. The WPT method, with short responses times was the most adequate signal processing method for real-time signal feature extraction in surface quality control. It is worth noting that multiple regression enabled the determination of the significant characterization parameters of signals, eliminating factors correlated to or with little significant impact on the response variable (*Ra*), which improved the fit of ANN. In comparison to the ANN technique, multiple regression permitted greater control of the method, without random events. Thus, the use of the ANN predictive modeling technique is recommended, but with the prior multiple regression analysis of significant variables to ensure only input variables providing significant information correlated to surface quality were used in the ANN.

## 5. Conclusions

In this study, multi-sensor data fusion for the real-time intelligent control of surface quality in CNC machining systems was examined. The sensors most widely used for the monitoring of the machining processes based on cutting force, vibration, and acoustic emission signals were analyzed. A total of four signal processing methods were compared: two global analysis of the signal in one single domain, time (TDA) or frequency (PSD), and two signal analysis methods in the combined time-frequency domain (SSA and WPT). Owing to the nonlinear and stochastic nature of the process, two predictive modeling techniques were evaluated: multiple regression and artificial neural networks. The information provided by each individual sensor and the fusion of sensors was evaluated in terms of predictive power, reliability, and response time.

In the analysis of individual signals, both cutting forces and vibration were correlated to surface finish, allowing for the building of models with the best predictive results. In general terms, the back force *F_p_*, responsible of the contact stability between tool and workpiece, was the cutting force component with the greatest influence on roughness. As for the vibration signals, the tool dynamic behavior in feed direction, registered by *a_f_*, was the component with most impact on surface finish. Regarding the fusion of signals, the combination of cutting force and vibration signals improved predictive power and reliability. No correlation was observed between the *AE* signal and surface finish in either the individual or the fusion of other signals, and both even worsened the results obtained.

In the global signal analysis methods in the single domain (TDA and PSD), frequency analysis exhibited the worst behavior, with low prediction and reliability levels in all the signals analyzed, proving to be inadequate for signal feature extraction on its own. The TDA method improved the results of the PSD, which reached the minimum levels required, but with prediction and reliability below the SSA and WPT methods, particularly for individual signals. In most cases, the TDA + PSD combination slightly improved the results of the TDA method, indicating this combination could be applicable according to the precision of system requirements. The analytical-computational cost of these methods was very low, with the best response times.

The decomposition of the signals into a time series with defined frequency ranges improved signal feature extraction. The WPT method maximized information extraction in cutting force and vibration signals, whereas the SSA method was only applicable to vibration signals. The SSA method obtained long response times, rendering it inadequate for real-time monitoring systems. However, the WPT had shorter response times, making it the most adequate signal feature extraction method for use in real-time surface quality control systems.

Frequency bandwidths with information correlated to surface finish in both cutting force and vibration signals, and the sampling frequencies for each type of signal for the monitoring of surface finish were determined. The excitation ranges of cutting force signals that most influenced surface finish were found at very low frequencies (0–156.25 Hz), whilst vibration signals were at high frequency (6250–9375 Hz). Moreover, the vibration frequencies most affecting surface finish were determined for the selection of cutting speeds that did not excite the system in these frequency ranges.

The ANN models obtained the best prediction and reliability results, and was the most efficient predictive technique, but the prior elimination of non-significant characterization parameters using multiple regression is recommended.

Furthermore, the most adequate characterization parameters were determined for signal feature extraction of the three types of signals analyzed. This allowed for optimum parametric signal characterization by monitoring only parameters correlated to surface finish.

The present study proposes a multi-sensor data fusion method for on-line monitoring of surface quality in automated machining systems. Multi-sensor data fusion enabled highly accurate and reliable real-time monitoring of surface finish similar to post-process methods. This allows for in-situ decision-making based on the predictions of intelligent surface quality control systems that analyze only on-line cutting process information, which translates into greater flexibility and the real-time detection of malfunctions. The results obtained underscored that the selection of sensors, signal feature extraction method, and predictive modeling technique were crucial aspects for optimizing monitoring systems.

## Figures and Tables

**Table 1:** Signal characterization parameters.

Features	Signal Components						
	*F_p_*	*F_f_*	*F_c_*	*a_p_*	*a_f_*	*a_c_*	*AE*
Mean	$X_{F_{p}}^{j}$	$X_{F_{f}}^{j}$	$X_{F_{c}}^{j}$	$X_{a_{p}}^{j}$	$X_{a_{f}}^{j}$	$X_{a_{c}}^{j}$	$X_{AE}^{j}$
Standard deviation	$\sigma_{F_{p}}^{j}$	$\sigma_{F_{f}}^{j}$	$\sigma_{F_{c}}^{j}$	$\sigma_{a_{p}}^{j}$	$\sigma_{a_{f}}^{j}$	$\sigma_{a_{c}}^{j}$	$\sigma_{AE}^{j}$
Peak to peak amplitude	$PP_{F_{p}}^{j}$	$PP_{F_{f}}^{j}$	$PP_{F_{c}}^{j}$	$PP_{a_{p}}^{j}$	$PP_{a_{f}}^{j}$	$PP_{a_{c}}^{j}$	$PP_{AE}^{j}$
Skewness	$S_{F_{p}}^{j}$	$S_{F_{f}}^{j}$	$S_{F_{c}}^{j}$	$S_{a_{p}}^{j}$	$S_{a_{f}}^{j}$	$S_{a_{c}}^{j}$	$S_{AE}^{j}$
Kurtosis	$K_{F_{p}}^{j}$	$K_{F_{f}}^{j}$	$K_{F_{c}}^{j}$	$K_{a_{p}}^{j}$	$K_{a_{f}}^{j}$	$K_{a_{c}}^{j}$	$K_{AE}^{j}$
Energy	$E_{F_{p}}^{j}$	$E_{F_{f}}^{j}$	$E_{F_{c}}^{j}$	$E_{a_{p}}^{j}$	$E_{a_{f}}^{j}$	$E_{a_{c}}^{j}$	$E_{AE}^{j}$
Shannon entropy	$SE_{F_{p}}^{j}$	$SE_{F_{f}}^{j}$	$SE_{F_{c}}^{j}$	$SE_{a_{p}}^{j}$	$SE_{a_{f}}^{j}$	$SE_{a_{c}}^{j}$	$SE_{AE}^{j}$

Where, j=t is time for TDA method, SSA $j=\lambda_{1},\ldots ,\lambda_{L}$ are number of the eigenvalues for SSA method, PSD j=f1,…,f4 are frequency ranges for PSD, and j=A,…,ADADA are frequency ranges (packets) for the WPT.

**Table 2:** Significant signal feature extraction of the optimum model obtained with WPT.

Feature	Frequency (Hz)	Sum of Sq (Type III)	*p*-Value
$X_{F_{p}}^{AAAA}$	0–156.25	3.30	$3.10\times 10^{-18}$
$\sigma_{F_{p}}^{DAAA}$	156.25–312.50	0.22	$1.39\times 10^{-2}$
$K_{F_{f}}^{DADA}$	937.50–1093.75	0.19	$2.17\times 10^{-2}$
$SE_{F_{c}}^{AAAA}$	0–156.25	5.37	$1.26\times 10^{-26}$
$SE_{a_{p}}^{DDA}$	6250–9375	1.42	$2.41\times 10^{-9}$
$\sigma_{a_{f}}^{DDA}$	6250–9375	3.82	$1.77\times 10^{-20}$
$PP_{a_{f}}^{DDA}$	6250–9375	0.14	$5.38\times 10^{-2}$
$SE_{a_{f}}^{DDA}$	6250–9375	1.41	$2.74\times 10^{-9}$
$X_{a_{f}}^{ADA}$	9375–12,500	1.45	$1.81\times 10^{-9}$
$SE_{a_{f}}^{ADA}$	9375–12,500	0.67	$3.03\times 10^{-5}$
$\sigma_{a_{c}}^{DDA}$	6250–9375	3.81	$1.90\times 10^{-20}$
$SE_{a_{c}}^{ADA}$	9375–12,500	1.22	$2.72\times 10^{-8}$

## References

[1] Hocheng H., Tseng H., Hsieh M., Lin Y. (2018). Tool wear monitoring in single-point diamond turning using laserscattering from machined workpiece. J. Manuf. Process..

[2] Barreiro J., Fernández-Abia A., González-Laguna A., Pereira O. (2017). TCM system in contour milling of very thick-very large steel plates based on vibration and AE signals. J. Mater. Process. Technol..

[3] Nouri M., Fussell B., Ziniti B., Linder E. (2015). Real-time tool wear monitoring in milling using a cutting condition. Int. J. Mach. Tool Manuf..

[4] Zhu K., Wong Y.S., Hong G.S. (2009). Multi-categorymicro-milling tool wear monitoring with continuous hidden Markov models. Mech. Syst. Signal Process..

[5] Khasawneh F., Munch E. (2016). Chatter detection in turning using persistent homology. Mech. Syst. Signal Process..

[6] Cao H., Lei Y., He Z. (2013). Chatter identification in end milling process using wavelet packets and Hilbert-Huang transform. Int. J. Mach. Tool Manuf..

[7] Siddhpura M., Paurobally R. (2012). A review of chatter vibration research in turning. Int. J. Mach. Tool Manuf..

[8] Liu Y., Li T., Liu K., Zhang Y. (2016). Chatter reliability prediction of turning process system with uncertainties. Mech. Syst. Signal Process..

[9] Segreto T., Karam S., Teti R. (2017). Signal processing and pattern recognition for surface roughness assessment in multiple sensor monitoring of robot-assisted polishing. Int. J. Adv. Manuf. Technol..

[10] Segreto T., Karam S., Teti R., Ramsing J. (2015). Cognitive decision making in multiple sensor monitoring of robot assisted polishing. Proc. CIRP.

[11] Nath C., Kapoor S., Srivastava A. (2017). Finish turning of Ti-6Al-4V with the atomization-based cutting fluid (ACF) spray system. J. Manuf. Process..

[12] Niaki F., Mears L.A. (2017). Comprehensive study on the effects of tool wear on surface roughness, dimensional integrity and residual stress in turning IN718 hard-to-machine alloy. J. Manuf. Process..

[13] Risbood K.A., Dixit U.S., Sahasrabudhe A.D. (2003). Prediction of surface roughness and dimensional deviation by measuring cutting forces and vibration in turning process. J. Mater. Process. Technol..

[14] Azouzi R., Guillot M. (1997). On-line prediction of surface finish and dimensional deviation in turning using neural network based sensor fusion. Int. J. Mach. Tool Manuf..

[15] Karam S., Teti R. (2013). Wavelet transform feature extraction for chip form recognition during, carbon steel turning. Proc. CIRP.

[16] Liao Z., Axinte D.A. (2016). On monitoring chip formation, penetration depth and cuttingmalfunctions in bone micro-drilling via acoustic emission. J. Mater. Process. Technol..

[17] Wang G., Guo Z., Yang Y. (2013). Force sensor based online tool wear monitoring using distributed Gaussian ARTMAP network. Sens. Actuator A Phys..

[18] Tangjitsitcharoen S. (2012). Analysis of Chatter in Ball End Milling by Wavelet Transform. Int. J. Ind. Manuf. Eng. (WASET).

[19] Kilundu B., Dehombreux P., Chiementin X. (2011). Tool wear monitoring by machine learning techiques and singular spectrum analysis. Mech. Syst. Signal Process..

[20] Yao Z., Mei D., Chen Z. (2010). On-line chatter detection and identification based on wavelet and support vector machine. J. Mater. Process. Technol..

[21] Salgado D.R., Alonso F.J. (2008). Analysis of the structure of vibration signals for tool wear detection. Mech. Syst. Signal Process..

[22] Diniz A., Liu J., Dornfeld D. (1992). Correlating tool life, tool wear and surface roughness by monitoring acoustic emission in finish turning. Wear.

[23] Andrade L.H., Mendes A., Vasconcelos W.L., Falco W., Rocha A. (2015). A new approach for detection of wear mechanisms and determination of tool life in turning using acoustic emission. Tribol. Int..

[24] Chen X., Li B. (2007). AE Method for Tool Condition Monitoring Based on Wavelet Analysis. Int. J. Adv. Manuf. Technol..

[25] Griffin J.M., Chen X. (2009). Multiple classification of the acoustic emission signals extracted during burn and chatter anomalies using genetic programming. Int. J. Adv. Manuf. Technol..

[26] Salgado D.R., Alonso F.J. (2007). An approach based on current and sound signals for in-process tool wear monitoring. Int. J. Mach. Tool Manuf..

[27] Carou D., Rubio E., Lauro C., Cardoso L., Davim P. (2017). Study based on sound monitoring as a means for superficial quality control in termittent turning of magnesium workpieces. Proc. CIRP.

[28] Weingaertner W.L., Schroeter R.B., Polli M.L., Oliveira Gomes J. (2006). Evaluation of high-speed end-milling dynamic stability through audio signal measurements. J. Mater. Process. Technol..

[29] Li X., Li H.-X., Guan X.-P., Du R. (2004). Fuzzy Estimation of Feed-Cutting Force From Current Measurement- A Case Study on Intelligent. IEEE Trans. Syst. Man. Cybern. C Appl. Rev..

[30] Al-Sulaiman F.A., Abdul M., Sheikh A.K. (2005). Use of electrical power for online monitoring of tool condition. J. Mater. Process. Technol..

[31] Rangwala S., Dornfeld D. (1990). Sensor integration using neural networks for intelligent tool condition monitoring. J. Eng. Ind..

[32] Dornfeld D., DeVries M. (1990). Neural networks sensor fusion for tool condition monitoring. CIRP Ann. Manuf. Technol..

[33] Noori-Khajavi A., Komaduri R. (1995). Frequency and time domain analyses of sensor signals in drilling-I. Correlation with drill wear. Int. J. Mach. Tool Manuf..

[34] Noori-Khajavi A., Komaduri R. (1995). Frequency and time domain analyses of sensor signals in drilling-II. Investigation on some problems associated with sensors integration. Int. J. Mach. Tool Manuf..

[35] Duro J., Padget J., Bowen C., Kim H., Nassehi A. (2016). Multi-sensor data fusion framework for CNC machining monitoring. Mech. Syst. Signal Process..

[36] Liu C., Wang C.F., Li Z.M. (2015). Incremental learning for online tool condition monitoring using Ellipsoid ARTMAP network model. Appl. Soft Comput..

[37] Segreto T., Simeone A., Teti R. (2012). Sensor fusion for tool state classification in nickel superalloy high performance cutting. Proc. CIRP.

[38] Shi D., Gindy N.N. (2007). Tool Wear Predictive Model Based on Least Squares Support Vector Machines. Mech. Syst. Signal Process..

[39] Segreto T., Karam S., Simeone A., Teti R. (2013). Residual stress assessment in Inconel 718 machining through wavelet sensor signal analysis and sensor fusion pattern recognition. Proc. CIRP.

[40] Rao P., Bhushan M., Bukkapatnam S., Zong Z., Byalal S., Beyca O., Fields A., Komanduri R. (2014). Process-machine interaction (PMI) modeling and monitoring of chemical mechanical planarization (CMP) process using wireless vibration sensors. IEEE Trans. Semiconduct. Manuf..

[41] Hessainia Z., Belbah A., Yallese M.A., Mabrouki T., Rigal J.F. (2013). On the prediction of surface roughness in the hard turning based on cutting parameters and tool vibrations. Measurement.

[42] Bhuiyan M.H., Choudhury I.A., Dahari M. (2014). Monitoring the tool wear, surface roughness and chip formation formation occurrences using multiple sensors in turning. J. Manuf. Syst..

[43] Guo Y.B., Ammula S.C. (2009). Real-Time acoustic emission monitoring for surface damage in hard machining. Int. J. Mach. Tool Manuf..

[44] Jeong H., Kim H., Lee S., Dornfeld D. (2006). Multi-sensor monitoring system in chemical mechanical planarization (CMP) for correlations with process issues. CIRP Ann. Manuf. Technol..

[45] Wang H., To S., Chan C.Y. (2013). Investigation on the influence of tool-tip vibration on surface roughness and its representative measurement in ultra-precision diamond turning. Int. J. Mach. Tool Manuf..

[46] Tangjitsitcharoen S. (2009). In-process monitoring and detection of chip formation and chatter for CNC turning. J. Mater. Process. Technol..

[47] Abouelatta O.B., Madl J. (2001). Surface roughness prediction based on cutting parameter and tool vibration in turning operation. J. Mater..

[48] Krolczyk G., Madura R., Nieslony P., Wieczorowski M. (2016). Surface morphology analysis of Duplex Stainless Steel (DSS) in Clean Production using the Power Spectral Density. Measurement.

[49] Salgado D.R., Alonso F.J., Cambero I., Marcelo A. (2009). In-process surface roughness prediction system using cutting vibrations in turning. Int. J. Adv. Manuf. Technol..

[50] García E., Núñez P.J. (2017). Surface roughness monitoring by singular spectrum analysis of vibration signals. Mech. Syst. Signal Process..

[51] Kunpeng Z., Yoke San W., Geok Soon H. (2009). Wavelet analysis of sensor signal for tool condition monitoring: A review and some new results. Int. J. Mach. Tool Manuf..

[52] García E., Núñez P.J. (2018). Analysis of cutting force signals by wavelet packet transform for surface roughness monitoring in CNC turning. Mech. Syst. Signal Process..

[53] García E., Núñez P.J. (2018). Application of the wavelet packet transform to vibration signals for surface roughness monitoring in CNC turning operations. Mech. Syst. Signal Process..

[54] Salgado D.R., Alonso F.J. (2006). Tool wear detection in turning operations using singular spectrum analysis. J. Mater. Process. Technol..

[55] Pandiyan V., Caesarendra W., Tjahjowidodo T., Tan H.H. (2018). In-process tool condition monitoring in compliant abrasive belt grinding process using support vector machine and genetic algorithm. J. Manuf. Process..

[56] Teti R., Jemielniak K., O’Donnel G., Dornfeld D. (2010). Advanced monitoring of machining operations. CIRP Ann. Manuf. Technol..

[57] Lauro C.H., Brandão L.C., Baldo D., Reis R.A., Davim J.P. (2014). Monitoring and processing signal applied in machining processes—A review. Measurement.

[58] Kirby E.D., Chen J.C. (2007). Development of a fuzzy-nets-based surface roughness prediction system in turning operations. Comput. Ind. Eng..

[59] Upadhyay V., Jain P.K., Mehta N.K. (2013). In Process prediction of surface roughness in turning of Ti–6Al–4V alloy using cutting parameters and vibration signal. Measurement.

[60] Ozel T., Karpat Y., Figueira L., Davim P.J. (2007). Modelling of surface finish and tool flank wear in turning of AISI D2 steel with ceramic wiper inserts. J. Mater. Process. Technol..

[61] Botcha B., Rajagopal V., Bukkapatnam S. (2018). Process-machine interactions and a multi-sensor fusion approach to predict surface roughness in cylindrical plunge grinding process. Proc. Manuf..

[62] Kannatey-Asibu E., Dornfeld D. (1982). A study of tool wear using statistical analysis of metal-cutting acoustic emission. Wear.

[63] Siddhpura A., Paurobally R. (2013). A review of flank wear prediction methods for tool condition. Int. J. Adv. Manuf. Technol..

[64] Frigieri E., Campos P., Paiva A., Balestrassi P., Ferreira J., Ynoguti C. (2016). A mel-frequency cepstral coefficient-based approach for surface roughness diagnosis in hard turning using acoustic signals and gaussian mixture models. Appl. Acoust..

[65] Pawade R.S., Soshi S.S. (2012). Analysis of acoustic emission signals and surface integrity in the high speed turning of inconel 718. J. Eng. Manuf. (Sage J.).

[66] Rao P., Bukkapatnam S., Beyca O., Kong Z., Komanduri R. (2014). Real-time identification of incipient surface morphology variations in ultraprecision machining process. J. Manuf. Sci. Eng..

[67] Masters T. (1993). Practical Neural Networks Recipes in C++.

